# Auto-detection of the coronavirus disease by using deep convolutional neural networks and X-ray photographs

**DOI:** 10.1038/s41598-023-47038-3

**Published:** 2024-01-04

**Authors:** Ahmad MohdAziz Hussein, Abdulrauf Garba Sharifai, Osama Moh’d Alia, Laith Abualigah, Khaled H. Almotairi, Sohaib K. M. Abujayyab, Amir H. Gandomi

**Affiliations:** 1 Department of Computer Science, Faculty of Information Technology, Middle East University, Amman, Jordan; 2 Department of Computer Sciences, Yusuf Maitama Sule University, Kofar Nassarawa, Kano, 700222 Nigeria; 3 Department of Computer Science, Faculty of Computes and Information Technology, University of Tabuk, 71491 Tabuk, Saudi Arabia; 4 Computer Science Department, Prince Hussein Bin Abdullah Faculty for Information Technology, Al Al-Bayt University, Mafraq, 25113 Jordan; 5 Department of Electrical and Computer Engineering, Lebanese American University, Byblos, 13-5053 Lebanon; 6 Hourani Center for Applied Scientific Research, Al-Ahliyya Amman University, Amman, 19328 Jordan; 7 Applied Science Research Center, Applied Science Private University, Amman, 11931 Jordan; 8 School of Engineering and Technology, Sunway University Malaysia, 27500 Petaling Jaya, Malaysia; 9 School of Computer Sciences, Universiti Sains Malaysia, 11800 Pulau Pinang, Malaysia; 10 Computer Engineering Department, Computer and Information Systems College, Umm Al-Qura University, 21955 Makkah, Saudi Arabia; 11 International College for Engineering and Management, 112 Muscat, Oman; 12 Faculty of Engineering and Information Technology, University of Technology Sydney, Ultimo, NSW 2007 Australia; 13 University Research and Innovation Center (EKIK), Óbuda University, Budapest, 1034 Hungary

**Keywords:** Computational biology and bioinformatics, Diseases, Health care

## Abstract

The most widely used method for detecting Coronavirus Disease 2019 (COVID-19) is real-time polymerase chain reaction. However, this method has several drawbacks, including high cost, lengthy turnaround time for results, and the potential for false-negative results due to limited sensitivity. To address these issues, additional technologies such as computed tomography (CT) or X-rays have been employed for diagnosing the disease. Chest X-rays are more commonly used than CT scans due to the widespread availability of X-ray machines, lower ionizing radiation, and lower cost of equipment. COVID-19 presents certain radiological biomarkers that can be observed through chest X-rays, making it necessary for radiologists to manually search for these biomarkers. However, this process is time-consuming and prone to errors. Therefore, there is a critical need to develop an automated system for evaluating chest X-rays. Deep learning techniques can be employed to expedite this process. In this study, a deep learning-based method called Custom Convolutional Neural Network (Custom-CNN) is proposed for identifying COVID-19 infection in chest X-rays. The Custom-CNN model consists of eight weighted layers and utilizes strategies like dropout and batch normalization to enhance performance and reduce overfitting. The proposed approach achieved a classification accuracy of 98.19% and aims to accurately classify COVID-19, normal, and pneumonia samples.

## Introduction

In December 2019, the city of Wuhan in China witnessed the onset of the COVID-19 outbreak caused by the SARS-CoV-2 virus, which subsequently spread globally to all seven continents^1,2^. Despite the production of COVID-19 vaccines, new variants such as Delta, Omicron, XBB, and BQ continue to emerge worldwide^3^. The virus responsible for this contagious illness is the severe acute respiratory syndrome coronavirus-2 (SARS-CoV-2)^3^ COVID-19, which appeared in 2019, is a novel disease with no prior historical record^4^. Initial data suggests that approximately 99% of positive cases experience mild symptoms, while the remaining 1% exhibit severe symptoms^5^.

On January 20, 2020, the United States of America reported its first seven COVID-19 infections, and by April 5, 2020, the nationwide cases reached approximately 300,000^6^. Coronaviruses are known to infect animals and can be transmitted to humans, leading to zoonotic diseases. Middle East respiratory syndrome coronavirus (MERS-CoV) and severe acute respiratory syndrome coronavirus (SARS-CoV) are two examples of coronaviruses causing severe respiratory diseases in humans^7^. As of April 24, 2023, the global tally of COVID-19 cases stood at 686,553,714, with 6,860,023 reported fatalities and 659,100,556 recoveries. Currently, there are 20,593,135 active cases, with 99.8% exhibiting mild symptoms and 0.2% classified as severe or critical^8^.

COVID-19 is a recent respiratory illness caused by the coronavirus that can significantly impact individuals unexpectedly. Common symptoms of the disease include fever, cough, difficulty in breathing, and sore throat^9,10^. Some patients may also experience symptoms such as nasal blockage, body aches, fatigue, and loss of taste^11^. The incubation period, or the time between infection and the onset of the earliest symptoms, is typically around 14 days^12^.

Real-time reverse transcription-polymerase chain reaction (RT-PCR) testing is the most widely used strategy for identifying and diagnosing COVID-19. It is considered the primary method for detecting the coronavirus infection^13^. In addition to RT-PCR, computed tomography (CT) scans and chest X-rays play a crucial role in the timely detection and management of contagious infections^14^. When an RT-PCR test yields a negative result, patients may undergo additional verification through radiological imaging to confirm or rule out the presence of the virus. This is necessary because RT-PCR testing has a relatively low sensitivity, ranging between 60 and 70%^15,16^. CT scans serve as an important screening tool alongside RT-PCR for identifying COVID-19, particularly in the early phase of the disease (around 0–2 days) when CT findings are more reliable than RT-PCR results^17,18^. Studies have shown that CT scans of patients who have recovered from COVID-19 pneumonia can reveal significant lung disease around 10 days after the onset of symptoms^19^.

COVID-19 presents certain radiological signatures that can be observed in chest X-rays, making it crucial for radiologists to carefully examine these images. However, the process of manual chest X-ray analysis can be time-consuming and may not always be accurate. Therefore, there is a need for automated methods to analyze chest X-rays^12^. The goal of the present study is to develop a computerized approach based on deep learning techniques for detecting COVID-19 cases using X-ray images^20^.

In recent years, machine learning (ML) has gained popularity in the field of medicine and has become a complementary tool for doctors^21^. Deep learning, a subfield of artificial intelligence (AI), is particularly well-suited for creating end-to-end models that can produce accurate results from input data without the need for manual feature extraction^22,23^. Deep learning techniques have been successfully applied to various medical tasks, such as identifying arrhythmia, classifying skin cancer, and diagnosing pneumonia using chest X-ray images^24–26^. While radiologists play a crucial role in medical diagnosis, AI technology can assist them in making accurate and efficient diagnoses^27^. Additionally, AI approaches can help address challenges related to the scarcity of RT-PCR test kits, testing costs, and result turnaround time^28–30^.

The COVID-19 pandemic initially presented challenges due to the ambiguity surrounding its diagnosis, mode of infection, and appropriate treatment. Given the large number of infections, it became necessary to leverage modern technology, such as artificial intelligence, to quickly identify the disease using chest X-rays. Timely diagnosis is crucial as any delay could result in patient fatalities.

The proposed approach in this study involves the development of a deep learning-based algorithm called a Custom Convolutional Neural Network (Custom-CNN) specifically designed for diagnosing COVID-19. Swift detection is essential due to the potential severity of COVID-19 if diagnosed late. Preprocessing of raw images plays a vital role in deep learning, and in this model, all X-ray images are resized to a standardized size of 224 × 224 pixels. The Custom-CNN model is constructed using network blocks and consists of eight weighted layers. Techniques like dropout and batch normalization are employed to enhance the algorithm's performance and reduce overfitting. The proposed model effectively addresses challenges such as vanishing and exploding gradients during the learning process. Stochastic gradient descent is utilized to train the model, with a cumulative batch size of 32 and a total of 30 training epochs.

The main contributions of this study are as follows:

1. We introduced a novel CNN model, Custom-CNN, for COVID-19 detection using chest X-ray images. To optimize the proposed network, several tests were conducted on various network hyperparameters, including split ratio, batch size, learning rates, and optimizer, which can impact the performance of the network.
2. A comparative study was performed using two public datasets to evaluate the proposed model against several state-of-the-art models, such as VGG16, VGG19, and others. The results demonstrated the superiority of the proposed algorithm over other algorithms.

The following sequence was used to display the remaining parts of the paper: Related works appear in section “Related works”. A summary of the dataset that was used and the suggested deep-learning approach are provided in section “Findings and interpretation”. The experimental design, the collected data, and the discussion are highlighted in section “Findings and interpretation”. Section “Conclusion” concludes the article and provides instructions for subsequent tasks.

## Related works

Given the rapid spread of COVID-19 and its significant impact on public health and the global economy, there is a pressing need to develop effective tools for assessing the presence of the disease. Recently, artificial intelligence (AI) techniques in conjunction with radiological technologies have been adopted to automatically diagnose COVID-19 in affected individuals.

Deep learning techniques have been particularly useful in analyzing chest X-rays quickly, as X-rays offer advantages such as low ionizing radiation exposure and portability compared to chest CT scans^31,32^. Ozturk et al.^33^ proposed a deep learning model with an end-to-end architecture that directly utilizes raw chest X-ray data for COVID-19 diagnosis, eliminating the need for manual feature extraction. This model was trained using a dataset of 125 chest X-ray images, highlighting the need for more precise diagnostic techniques. One challenge in interpreting chest radiographs is the early detection of COVID-19 infection, as ground glass opacity (GGO), a common finding in COVID-19 cases, may have low sensitivity. However, well-trained deep learning models can focus on details that may be imperceptible to the human eye, potentially addressing this limitation.

Hemdan et al.^34^ introduced COVIDX-Net, an AI model capable of automatically detecting COVID-19 positivity in patients based on chest X-ray images. It achieved a classification accuracy of 91% when tested on a dataset of 75 individuals, with 25 confirmed positive cases and 50 negative cases. Sethy and Behera^35^ utilized a pre-trained transfer technique called ResNet-50 to extract imaging features from COVID-19 patients and employed support vector machines (SVM) for classification, achieving a classification accuracy score of 95.348%. Wang and Wong^36^ developed COVID-Net, a deep learning model for COVID-19 detection, which demonstrated a classification accuracy of 92.4% for distinguishing between normal cases, non-COVID pneumonia, and COVID-19 patients. Additionally, Ozturk et al.^33^ presented a novel model for automatic COVID-19 diagnosis using raw chest X-ray images, achieving high accuracy (98.08%) for both multi-class classification (COVID vs. No-Findings vs. Pneumonia) and binary classification (COVID vs. No-Findings). In another study, the YOLO real-time object detection system was used, employing the DarkNet model with 17 convolutional layers, each having a separate filter^33^.

Narayan Dasa et al.^37^ utilized chest X-ray images to develop a new deep-transfer learning-based technique for automatic detection of coronavirus disease. They suggested that these techniques can be used to leverage the strengths of networks trained on large datasets and modify the parameters of already trained networks on small datasets. However, there are limitations on how these techniques can be applied to X-rays.

Apostolopoulos and Mpesiana^38^ employed transfer learning to overcome the lack of images typically required to build a reliable CNN model. They used two datasets to support their findings. The first dataset consisted of 1427 X-ray images, including 224 COVID-19 cases, 700 cases of common bacterial pneumonia, and 504 normal cases. The second dataset comprised 1442 images, with 504 normal cases, 714 cases of viral and bacterial pneumonia, and 224 confirmed COVID-19 cases. Comparative analysis of various CNN models, including Xception, VGG19, Inception, MobileNet v2, and Inception ResNet v2, resulted in the best performance. When comparing MobileNet v2 and VGG19, the accuracy, sensitivity, and specificity were 98.75% for the 2-class classification and 93.48% for the 3-class classification, with sensitivity and specificity values of 92.85% and 98.75%, respectively.

In a similar context, Nishio et al.^39^ employed transfer learning with CNN models pre-trained on large datasets to enhance the reliability and robustness of models trained on smaller datasets. The models they used included ResNet-50, VGG16, MobileNet, EfficientNet, and DenseNet-12. They specifically utilized the VGG16 model as a deep learning model for their proposed approach. Various data augmentation techniques, such as shifting, flipping, mixing up, rotating, random image cropping, and patching, were employed to compensate for the limited amount of data available and improve the model's performance. The method achieved a sensitivity of 90% for COVID-19 pneumonia and an accuracy of 83.6% when compared to non-COVID-19 pneumonia cases and healthy individuals.

Li and Zhu^40^ developed the COVID-Xpert technology, which leveraged chest X-ray radiography imaging properties from a larger dataset of pneumonia and normal cases, refined with a small number of COVID-19 patients, to identify coronavirus cases using CNN models. They utilized the DenseNet-121 deep neural network architecture for pre-training their models, addressing the lack of COVID-19 cases and improving the model's effectiveness. Instead of using a more general dataset like ImageNet, they trained the DenseNet-121 model on closely related datasets, specifically chest X-ray photographs with 108,948 samples. They tested the proposed model using 555 chest X-ray images categorized into three classes: 185 normal, 185 pneumonia, and 185 COVID-19 images. Their classification accuracy of 88.9% achieved an area under the ROC curve of 0.973.

Oh et al. ^41^ tackled the issue of the absence of specialized COVID-19 chest X-ray images by developing a patch-based CNN approach for coronavirus assessment with a manageable number of trainable parameters. Their suggested model included a pre-processing step to normalize data heterogeneities and bias, a segmentation network to extract the lung region, and a classification network for patch-by-patch training and inference. The model achieved sensitivities of 90%, 93%, and 100% for normal, pneumonia, and COVID-19 images, respectively, with corresponding precision values of 95.7%, 90.3%, and 76.9%.

Nigam et al.^42^ employed well-known deep learning architectures, including Xception, NASNet, VGG16, DenseNet121, and EfficientNet, in their work. The accuracies obtained for these models were 79.01%, 89.96%, 88.03%, 85.03%, and 93.48%, respectively.

To address the similarities between pneumonia and COVID-19 variables in chest X-rays, Khuzani^43^ employed a dimensionality reduction method with a neural network classifier (CXR). The Kernel-Principal Component Analysis (PCA) technique was used to decrease the dimension of the feature space, and a total of 420 images (120 normal, 120 coronavirus, and 120 non-coronavirus pneumonia images) were collected to create the classifier.

Gour^44^ utilized X-ray and CT images to develop an automated COVID-19 detection system using layered ensemble convolutional neural networks. Multiple layered convolutional neural network sub-models were employed to diagnose COVID-19 based on these images. A softmax classifier was used to stack the submodels from the Xception and VGG19 models. To demonstrate the discriminating power of the stacked CNN model, 4645 CT scans from 65 patients were collected. Out of these, 2249 images were found to have COVID-19, while 2396 were assessed as being in excellent health. The stacked CNN model achieved a true positive rate of 97.62% for multi-class classification.

For the categorization of X-ray images in diagnosing COVID-19, Karac^45^ utilized pre-trained VGGCOV19-NET, VGG19, deep CNN models, and the Cascade model with the YOLOv3 detection technique. The accuracy of the models was evaluated using metrics such as the confusion matrix, ROC, precision, specificity, and F1-score, along with a fivefold cross-validation technique. The Cascade VGGCOV19-NET model achieved an overall accuracy of 99.84% for the binary class dataset. Compared to VGG19 and VGGCOV19-NET, the Cascade VGGCOV19-NET model exhibited a higher accuracy rate.

Medhi^46^ proposed a rapid deep CNN approach for identifying coronavirus-infected patients from X-ray images. The John Hopkins University-produced Kaggle dataset, which includes data from 150 COVID-19 patients gathered in Wuhan, was utilized to assess the effectiveness of the suggested CNN strategy. The results of the proposed deep CNN method revealed an overall accuracy of 93%.

To classify coronavirus X-ray images, Abbas^47^ examined the Decompose, Transfer, and Compose (DeTraC) models. The DeTraC model employed a class decomposition method to analyze the class boundaries and handle X-ray image irregularities. The results demonstrated the DeTraC model's capability to categorize COVID-19 images, achieving a performance accuracy of 93.1% in separating COVID-19 X-ray images from background images.

Bargshady^48^ utilized a large dataset consisting of Coronation X-ray and CT chest images. The generative adversarial network (GAN) method was employed in conjunction with the trained semi-supervised CycleGAN (SSA-CycleGAN) model. The images were enhanced using the model during training. The proposed Inception-CycleGAN model achieved an overall accuracy of 94.2%, a mean absolute error of 0.16, a mean squared error of 0.27, and an ROC-Area under the Curve of 92.2%.

Kanwal^49^ proposed a classification model (2dCNN-BiCuDNNLSTM) for early identification of fatal coronavirus disease. The technique combined a bidirectional CUDA Deep Neural Network Long Short-Term Memory with a double Convolutional Neural Network (CNN) (BiCuDNNLSTM). The suggested model successfully distinguished between COVID-19 patients and images of healthy chest X-rays (2dCNN and BiCuDNNLSTM layers). The proposed approach was evaluated on 6863 X-ray images, and the results demonstrated the effectiveness of the suggested model with a total performance accuracy of 93%.

Sahin^50^ created a CNN model for automatic COVID-19 detection using 13,824 chest X-ray images. The suggested CNN model employed various pre-trained models and model structures, including MobileNetv2 and ResNet50, to differentiate COVID cases from typical X-ray images. According to the experimental findings, the CNN model achieved an overall accuracy of 96.71% and an F1-score of 91.89% in categorizing COVID-19 cases. The outcomes of this model surpassed several existing high-end techniques.

Authors in ^51^ examines the impact of an unbalanced dataset on the performance of active learning techniques for chest X-ray image classification. The study evaluates various scoring functions and sampling strategies to prioritize data for labeling. Scoring functions include model uncertainty based on confidence and margin scoring, as well as expected model change. Sampling strategies include top-N sampling, K-nearest neighbors sampling, core-set sampling, and mixture sampling. The results show that the choice of scoring function and sampling strategy significantly affects the performance of active learning algorithms in the context of an unbalanced dataset. A comparison of the techniques on a 40% COVID-19 dataset shows that core-set and margin scoring achieve the highest accuracy, with 89.86% and 90.87% respectively, while confidence and expected model change achieve slightly lower accuracy.

Sharma et al.^52^ proposed a shallow architecture called Convolutional Capsule Network (Conv-CapsNet) to detect COVID-19 infections. Combining capsule networks’ ability to understand spatial information with convolutional layers for feature extraction, the model has 23 M parameters and requires fewer training samples. Despite the limited training data, the Conv-CapsNet achieves an average accuracy of 96.47% for multi-class and 97.69% for binary classification on fivefold cross-validation. The modifications include adding four convolutional layers to enhance feature extraction, resizing images to 150 × 150 pixels, and applying Contrast Limited Adaptive Histogram Equalization (CLAHE) for improved data suitability and performance.

Nikolaou et al. in^53^ focused on developing a hybrid convolutional neural network (CNN) for differentiating COVID-19 from other viral pneumonia and normal lungs using chest X-ray images. The CNN is based on the EfficientNetB0 model, which has fewer parameters and requires less computational power compared to other models. A dense layer is added on top of the baseline model for feature extraction. The CNN achieves high accuracy in differentiating COVID-19 from normal lungs and other viral pneumonia. Data augmentation and dropout techniques are used to address overfitting. The CNN shows promise in assisting clinicians with accurate diagnostic decisions and supporting chest X-rays as a screening tool for early COVID-19 diagnosis. The model achieved a 95% accuracy, 90% sensitivity, and 97% specificity in distinguishing COVID-19 cases from normal lungs.

Aslan et al.^29^ focuses on the early diagnosis of COVID-19 using machine learning methods applied to chest X-ray images. A dataset of 15,153 X-ray images belonging to three classes (COVID-19, Normal, and Viral Pneumonia) was used. The dataset underwent preprocessing and was then fed into various classification methods, including Cubic SVM, LD, QD, Ensemble, KNB, and KNN Weighted. The Local Binary Pattern (LBP) texture operator was applied for feature extraction. The results showed that using LBP improved the accuracy from 94.1 to 98.05%, with the Cubic SVM method performing the best. The study demonstrates the effectiveness of LBP feature extraction in improving classification performance for COVID-19 detection in chest X-ray images.

Authors in^54^ proposed a model based on the VGG16 architecture, fine-tuned with custom layers, for multiclass classification of chest X-ray images. The model achieved a 98% accuracy, 98% precision, 96% recall, and 97% F1 score on the test dataset. The area under the receiver operating characteristic curve was 0.99 for multiclass classification. The proposed model can be valuable for preliminary diagnosis, particularly during heavy workloads. The study explored image preprocessing, augmentation, and VGG16-specific techniques to enhance image features.

A CapsNet model called CapsNetCovid was developed for COVID-19 diagnosis using CT and X-ray images^55^. It achieved high classification accuracy, precision, sensitivity, and F1-score of 99.929%, 99.887%, 100%, and 99.319% respectively for CT images, and 94.721%, 93.864%, 92.947%, and 93.386% respectively for X-ray images. CapsNetCovid outperformed CNN, DenseNet121, and ResNet50 models for standard and augmented CT and X-ray images. It showed better resistance to image rotations and transformations compared to other models. Data augmentation improved the CapsNet's performance, and future research can focus on enhancing its generalization and robustness for multi-class classification problems. The study aims to aid accurate COVID-19 diagnosis by medical professionals.

Researchers in^56^ used deep learning algorithms, VGG16 and ResNet50, to extract features from chest X-ray images and classify them into viral pneumonia, normal, and COVID-19 categories. The models achieved average accuracies of 89.34% (VGG16) and 91.39% (ResNet50) for COVID-19 detection. Larger datasets are beneficial for improving accuracy when using deep learning. The recommended system involves dataset creation, preprocessing, CNN implementation, output classification, loss calculation, parameter adjustment, and repetition for all datasets and epochs. VGG16 and ResNet50 models were effective for COVID-19 classification, with ResNet50 performing better.

Several machine learning (ML) models have been trained and used in the literature for COVID-19 detection. Transfer learning has been employed using various pre-trained models, including COVIDX-Net, ResNet-50, MobileNetv2, DarkNet, Inception, Xception, Inception ResNet v2, VGG16, ResNet-50, MobileNet, DenseNet-121, Cascade VGGCOV19-NET, EfficientNet, Xception, VGGCOV19-NET, DeTraC, NASNet, and CycleGAN. These pre-trained models have demonstrated accuracy levels ranging from 79 to 93%. Additionally, some authors have developed their own models, such as the 2dCNN-BiCuDNNLSTM and BiCuDNNLSTM models, which have shown higher performance results, reaching an accuracy of 96.71%. It is worth noting that the accuracy of the models tends to decrease when applied to a larger set of X-ray images compared to achieving high accuracy with a small number of photos. Binary classifiers that performed exceptionally well and achieved accuracy levels surpassing 99% in many earlier works showed lower overall accuracy when classifying three groups (coronavirus, healthy, and pneumonia patients).

## Methods and material used

### Dataset characterization

**Figure 1:**
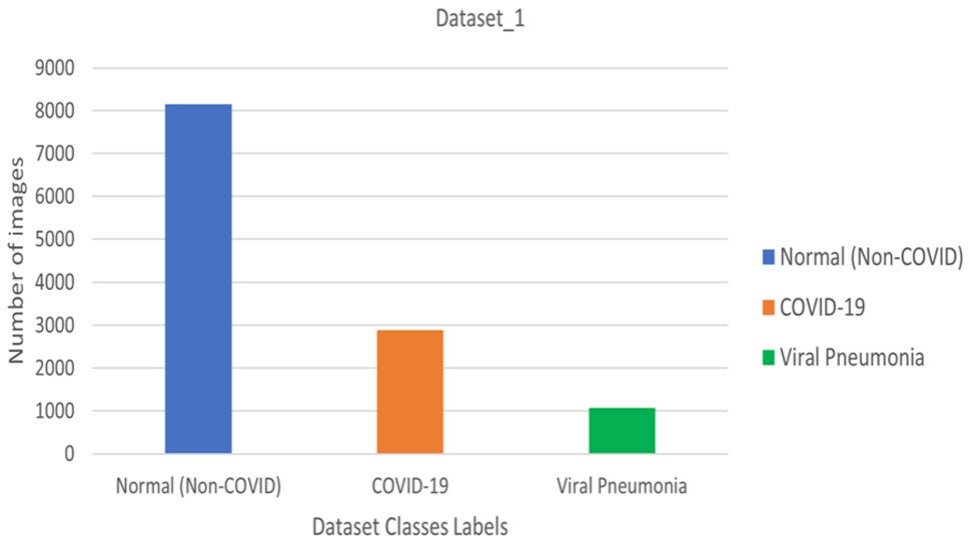
Multi-Class dataset description.

**Figure 2:**
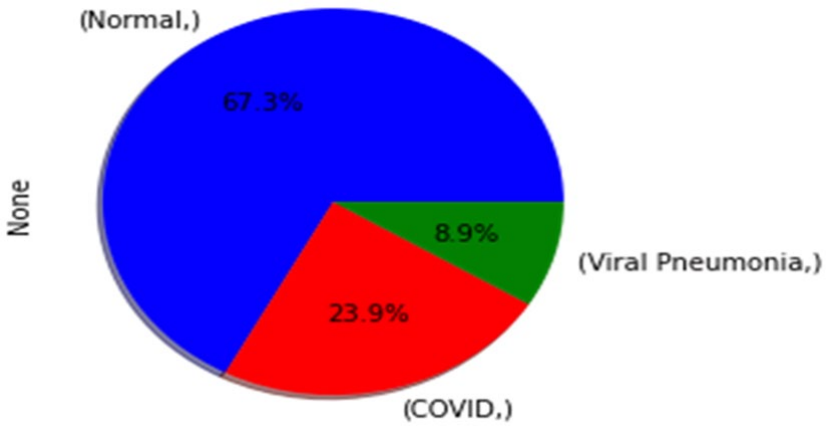
Illustration of the percentage of each class.

Two chest X-ray datasets were downloaded from free resources such as Kaggle to test and train the intended model. It is crucial to properly validate the performance of the suggested models using samples from the same category under assessment. The first dataset, referred to as dataset_1, is presented in Fig. 1 and consists of three categories: normal, coronavirus-positive, and viral pneumonia. The distribution of each class is illustrated in Fig. 2. Dataset_1 was developed by a group of scholars from Malaysia, Bangladesh, Pakistan, and Qatar and obtained from Kaggle^57^. It includes a total of 15,153 chest X-ray images, with 3,616 coronavirus-positive images, 1,345 viral pneumonia images, and 10,192 normal images. Figure 4 shows an example of dataset_1, depicting the three classifications: COVID-19, non-COVID-19 (Normal), and viral pneumonia^58^.

**Figure 3:**
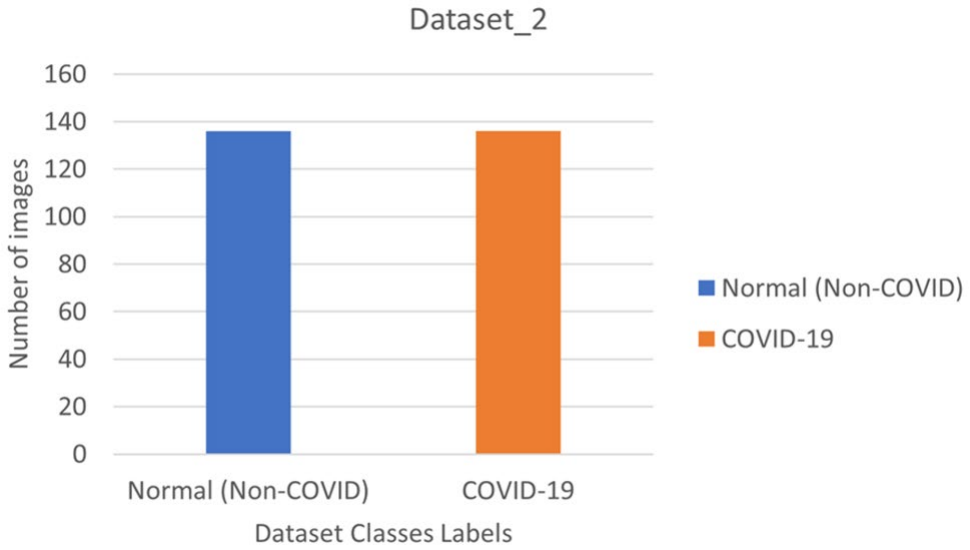
Binary class description.

The second dataset, labeled dataset_2, is represented in Fig. 3 and consists of two primary classes: normal and coronavirus-positive. Dataset_2 includes a total of 340 chest X-rays, evenly distributed between normal and coronavirus images. This dataset can be found on GitHub^59^, and each class contains 170 images after equal distribution^60^. The main objective of the study is to utilize these datasets to conduct efficient scientific research on COVID-19 to aid in combating the pandemic.

For training the suggested Custom-CNN model, 80% of the total chest X-ray images were used, while 20% were reserved for testing. Table 1 provides a detailed description of the normal (non-coronavirus), coronavirus, and viral pneumonia categories, along with the percentages of dataset division.

**Table 1:** Dataset descriptions for the proposed model training and testing (80% and 20%).

Datasets (Images #)	Training/Categories				Testing/Categories			
	Training total	Normal (Non-coronavirus)	Coronavirus	Viral Pneumonia	Testing total	Normal (Non-coronavirus)	Coronavirus	Viral Pneumonia
dataset_1 (15,153)	12,123	8154	2893	1076	3030	2038	723	269
dataset_2 (340)	272	136	136	–	68	34	34	–

### Pre-processing

**Figure 4:**
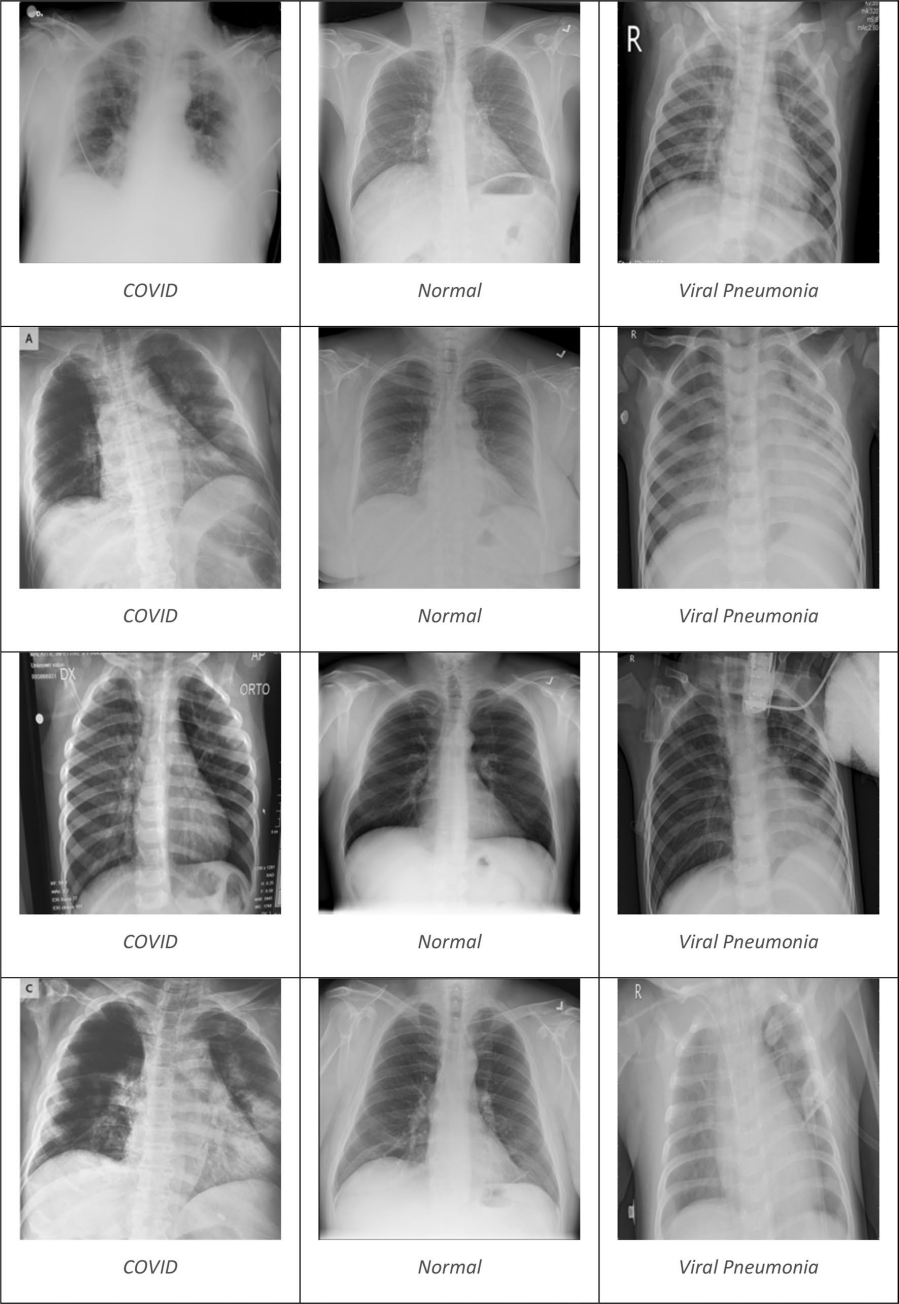
A graphical illustration of Coronavirus, Non-coronavirus (Normal), and Viral Pneumonia images.

Preprocessing is a crucial stage in deep learning techniques. It is an essential requirement for developing a model that yields good performance in the Convolutional Neural Network system used for COVID-19 detection. The input images have varying sizes in terms of width and length, necessitating the resizing of the input images. In this study, the two datasets consist of images with different dimensions (Width * Length). Therefore, the images were resized to the same dimensions for both datasets (224 * 224 pixels). A classification task was conducted, involving two and three categories, which were evaluated in this research study (Fig. 4).

### Convolutional neural network (Custom-CNN)

To handle complex real-world scenarios while maintaining sufficient accuracy, numerous modifications have been made to CNN structures^61^. This section, which examines the structure of the proposed solution, presents the main argument of this research report. The CNN architecture of the proposed solution stands out due to the combination of methods used to construct this multi-level complex network. The development of the network and the arrangement of its building elements, including pooling, convolution, flattening, and fully connected layers, are collectively referred to as the “mix” in this context. In order for this algorithm to identify whether X-ray images of the patients under investigation depict health or disease, it requires access to the underlying features hidden within the X-ray images.

**Figure 5:**
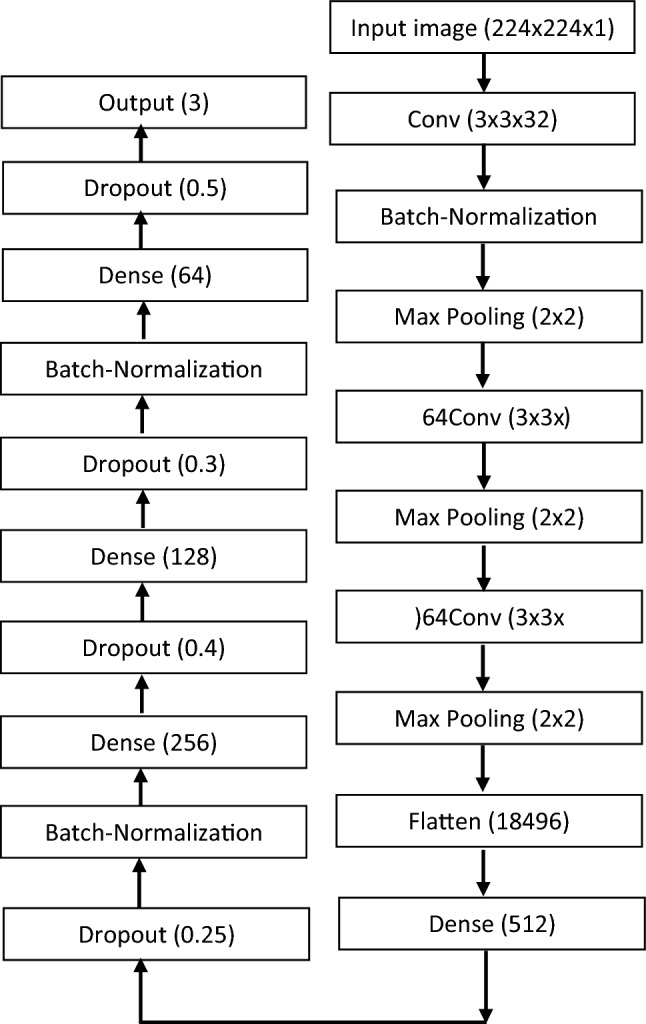
COVID-Custom-CNN architecture.

As shown in Fig. 5, our suggested Custom-CNN model comprises eight weighted layers, with the first three being convolutional and the remaining five being fully connected. The initial convolutional layer filters the input image, which is 224 × 224 pixels, using 32 kernels of size 3 × 3, with a stride of one pixel and "valid" padding. The size of the subsequent layers in the CNN sequence is the same as the Max-pooling layer, which has a size of 2 × 2. However, the input size to the second and third convolutional layers differs from the first layer. The second and third convolutional layers each utilize 64 kernels of size 3 × 3, with a stride of one pixel and “valid” padding. Consequently, the input size for the third layer changes to 36 × 36 × 64, and for the second layer, it changes to 111 × 111 × 64. All three layers apply the ReLU activation function to introduce nonlinearity to their outputs. The output of the third convolutional layer, with a size of 17 × 17 × 64, is flattened into a 1-dimensional array of size 1 × 18,496.

The remaining levels of the Custom-CNN model in this example consist of fully connected layers. The first fully connected layer has 2 neurons, the second has 256 neurons, the third has 128 neurons, and the fourth has 64 neurons. The ReLU activation function is utilized in these fully connected layers to nonlinearize their outputs. The output is then fed into a three-way Softmax function, which generates probabilities for the three class labels in the study: normal, COVID-19 positive, and viral pneumonitis. This final output represents the fully connected layer in contrast to the previous layers.

Due to the proposed algorithm having approximately ten million trainable parameters, the issue of overfitting arises, where the model performs better on the training data than on the test data. To address this problem, various well-known strategies were employed, including data augmentation, ℓ1 and ℓ2 regularizations, batch normalization, early stopping, and dropout. Among these strategies, dropout and batch normalization proved effective in improving the algorithm's performance and reducing overfitting. However, data augmentation, ℓ1 and ℓ2 regularization, and early stopping had limited impact in the conducted studies.

Dropout is a technique where each neuron has a probability of being temporarily “dropped out” during training, excluding the input and output neurons. This means that the neuron's contribution is temporarily ignored during training but can be effective in subsequent steps. In this study, the initial dropout was set to a probability of 0.25 after the first fully connected layer, followed by subsequent dropouts with probabilities of 0.4, 0.3, and 0.5 after the second, third, and fourth fully connected layers, respectively.

Batch normalization is a method used to normalize input values or bring numerical data to the same scale without altering its structure. It greatly reduces the number of training epochs required for training deep networks and stabilizes the learning process. In the proposed network, batch normalization was applied to the inputs of the second convolutional layer, the second fully connected layer, and the fourth fully connected layer.

It is worth noting that the learning process of the suggested network mitigated the effects of well-known issues such as vanishing gradients and exploding gradients. Exploding gradients can cause exponential growth, resulting in significant weight updates in multiple layers and causing the algorithm to diverge. Vanishing gradients occur when the algorithm descends to lower layers, and the gradients become extremely small. These problems are well-recognized, and there are established methods that focus on network weight initialization, such as Glorot and Bengio and He et al., which were utilized in all layers of the proposed network. Additionally, the batch size was set to 32 examples, and the model was trained using stochastic gradient descent for a total of 30 epochs. The summarized details of the proposed Custom-CNN model can be found in Table 2.

**Table 2:** Summary of the custom-CNN model.

Model: “sequential"		
Layer (type)	Output shape	Param #
conv2d (Conv2D)	(None, 222, 222, 32)	320
batch_normalization (BatchNo)	(None, 222, 222, 32)	128
max_pooling2d (MaxPooling2D)	(None, 111, 111, 32)	0
conv2d_1 (Conv2D)	(None, 109, 109, 64)	18,496
batch_normalization (BathNo)	(None, 109, 109, 64)	256
max_pooling2d_1 (MaxPooling 2D)	(None, 36, 36, 64)	0
conv2d_2 (Conv2D)	(None, 34, 34, 64)	36,928
max_pooling2d_2 2D) (MaxPooling	(None, 17, 17, 64)	0
flatten (Flatten)	(None, 18,496)	0
dense (Dense)	(None, 512)	9,470,464
dropout (Dropout)	(None, 512)	0
batch_normalization (BathNo)	(BatchNo) (None, 512)	2048
dense_1 (Dense)	(None, 256)	131,328
dropout_1 (Dropout)	(None, 256)	0
dense_2 (Dense)	(None, 128)	32,896
dropout_2 (Dropout)	(None, 128)	0
batch_normalization_2 (BatchNo)	(None, 128)	512
dense_3 (Dense)	(None, 64)	8256
dropout_3 (Dropout)	(None, 64)	0
dense_4 (Dense)	(None, 3)	195
Total params: 9,701,827		
Trainable params: 9,700,355		
Non-trainable params: 1472		

## Findings and interpretation

This section demonstrates the efficiency of the suggested Custom-CNN model in classifying COVID-19, pneumonia, and normal chest X-ray images for dataset_1 and dataset_2. Following the training process, the performance parameters based on the confusion matrix, including accuracy, precision, recall/sensitivity, F1-score, and test loss, are reported using the terms true positive (TP), true negative (TN), false positive (FP), and negative rates (FN).

The expected and actual classifications of coronavirus X-ray images (i.e., pneumonia, normal, and coronavirus) are presented in Table 3 as a confusion matrix. This provides a detailed representation of the pre-processing and evaluation metrics for the two datasets. Section “Evaluation of the Custom-CNN using dataset_1” discusses dataset_1, while section “Evaluation of the Custom-CNN using dataset_2” focuses on dataset_2.

**Table 3:** Confusion matrix.

	Positive	Negative
Positive	TP	FN
Negative	FP	TN

The effectiveness of the Custom-CNN method can be evaluated using various metrics. In this study, the proposed model was assessed using the following metrics: accuracy, precision, recall/sensitivity, F1-score, and test loss, which were determined using the confusion matrix.

Accuracy refers to the overall performance measurement, specifically the total number of correct predictions made.

$$Accuracy = \frac{{\left( {TP + TN} \right)}}{{\left( {TP + TN + FP + FN} \right)}}$$ .

Precision refers to the proportion of correctly predicted positive observations out of the total predicted positive observations.

$$Precision = \frac{TP}{{\left( {TP + FP} \right)}}$$.

Recall (sensitivity) refers to the proportion of correctly predicted positive observations to all observations in the current actual class.

$$Recall \left( {{\text{sensitivity}}} \right) = \frac{TP}{{\left( {TP + FN} \right)}}$$.

The F1 score refers to the metric that provides a single score that balances both precision and recall concerns into one number.

$$F1 - Score = 2* \frac{{\left( {Recall* Precision} \right)}}{{\left( {Recall + Precision} \right)}}$$.

Based on the previously specified criteria, the classification method assesses the effectiveness of the suggested strategy. The results of applying the proposed procedures to dataset_1 and dataset_2 are described in the following subsections.

### Evaluation of the Custom-CNN using dataset_1

Based on various hyperparameter adjustments, we investigated the performance of the proposed Custom-CNN model on COVID-19 images. For instance, we examined the model’s performance regarding batch sizes, acquisition rate, and pre-trained network designs. In the first set of experiments, we evaluated the effectiveness of the suggested model in comparison to a CNN pre-trained network.

**Figure 6:**
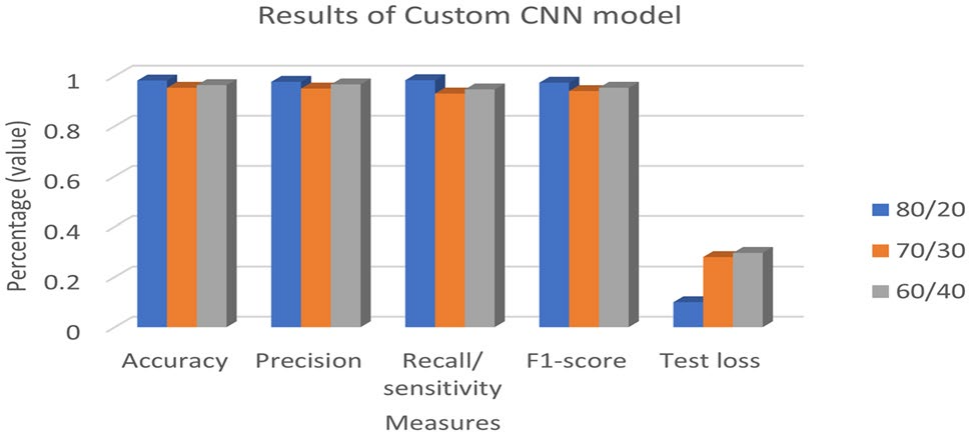
Results of a Custom-CNN model with various splitting ratio percentages.

Table 4 and Fig. 6 present the results for three split ratios: 80/20, 70/30, and 60/40. It was observed that the 80/20 split ratio consistently yielded higher results for accuracy, precision, recall, F1-score, and test loss, with values of 0.9819, 0.9767, 0.9833, and 0.073, respectively, compared to the 70/30 and 60/40 split ratios. The acquired data demonstrated that, based on all the performance indicators, an 80/20 split ratio produced the best outcomes.

**Table 4:** Results of a custom-CNN model with various splitting ratio percentages.

Split ratio (train/test)	Accuracy	Precision	Recall/sensitivity	f1-score	Test loss
**80/20**	**0.9819**	**0.9767**	**0.9833**	**0.9733**	**0.073**
70/30	0.9530	0.95	0.93	0.94	0.27933
60/40	0.9637	0.9666	0.9466	0.9533	0.2959

Significant values are in bold.

**Figure 7:**
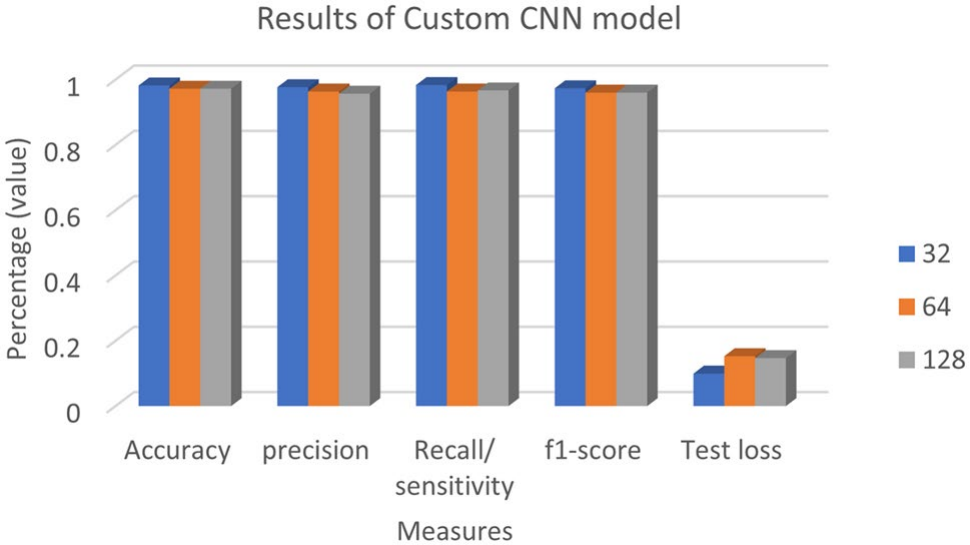
Results of a Custom-CNN model using various batch sizes.

The effectiveness of the suggested Custom-CNN model was further examined in a second series of tests, focusing on various batch sizes. Table 5 and Fig. 7 present the results for three applicable batch sizes: 32, 64, and 128. It was observed that a batch size of 32 consistently yielded higher classification results for accuracy, precision, recall, F1-score, and test loss compared to batch sizes of 64 and 128. The classification scores for accuracy, precision, recall, and F1-score were 0.9819, 0.9767, 0.9833, and 0.073, respectively, for a batch size of 32. The collected data provided evidence that a batch size of 32 produced the best results across all performance indicators.

**Table 5:** Results of a Custom-CNN model using various batch sizes.

# Batch size	Accuracy	precision	Recall/ sensitivity	f1-score	Test loss
**32**	**0.9819**	**0.9767**	**0.9833**	**0.9733**	**0.073**
64	0.9719	0.963333	0.963333	0.96	0.1528
128	0.9720	0.956667	0.966667	0.96	0.1469

Significant values are in bold.

The effectiveness of the suggested Custom-CNN model was further examined experimentally by considering different learning rate values. Table 6 displays 10 learning rate values (0.001, 0.002, 0.003, 0.004, 0.005, 0.0001, 0.0002, 0.0003, 0.0004, and 0.0005) and reveals that higher classification results of 0.9819, 0.9767, 0.9833, 0.9733, and 0.073 were achieved with a learning rate value of 0.001 for accuracy, precision, recall, F1-score, and test loss, respectively, compared to the other learning rate values. These results confirm that a learning rate of 0.001 consistently yields the best performance across all the measured criteria.

**Table 6:** Results of Custom-CNN model with various learning rates.

Learning rate	Accuracy	precision	Recall/sensitivity	f1-score	Test loss
**0.001**	**0.9819**	**0.9767**	**0.9833**	**0.9733**	**0.073**
0.002	0.9489	0.9367	0.9367	0.9367	0.1644
0.003	0.9506	0.9433	0.93	0.9367	0.1998
0.004	0.9588	0.9433	0.9567	0.95	0.1801
0.005	0.9638	0.9567	0.96	0.9567	0.1742
0.0001	0.9687	0.9567	0.9667	0.96	0.1211
0.0002	0.9638	0.9733	0.95	0.96	0.1703
0.0003	0.9259	0.94	0.87	0.9	0.3394
0.0004	0.9325	0.91	0.95	0.9267	0.2961
0.0005	0.9292	0.9233	0.9233	0.92	0.2603

Significant values are in bold.

**Figure 8:**
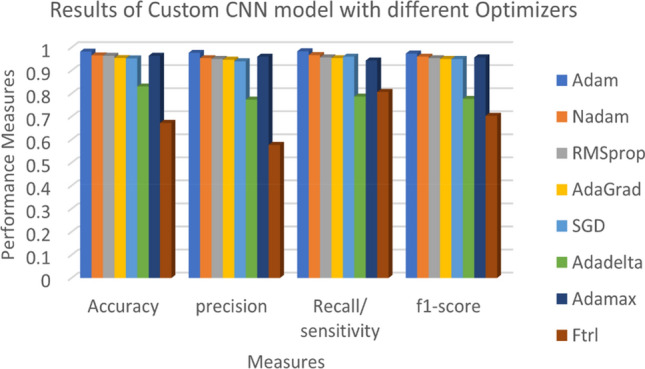
Custom-CNN model outcome with different optimizers.

Experimentally, the effectiveness of the proposed Custom-CNN model was further investigated by considering various CNN optimizers. Table 7 and Fig. 8 present the results of experiments conducted using eight CNN optimizers, namely Adam, Nadam, RMSprop, AdaGrad, SGD, Adadelta, Adamax, and Ftrl. The experimental results indicate that the highest classification results of 0.9819, 0.9767, 0.9833, 0.9733, and 0.073 were achieved with the Adam optimizer for accuracy, precision, recall, F1-score, and test loss, respectively, surpassing the classification performance obtained by the other optimizers. The obtained data demonstrate that the Adam optimizer consistently delivered the best outcomes across all performance measures.

**Table 7:** Results of Custom-CNN model with different optimizers.

Optimizer name	Accuracy	Precision	Recall/ sensitivity	f1-score	Test loss
Adam	**0.9819**	**0.9767**	**0.9833**	**0.9733**	**0.073**
Nadam	0.9654	0.9533	0.9667	0.96	0.1409
RMSprop	0.9638	0.95	0.9567	0.9533	1.545
AdaGrad	0.9539	0.9467	0.9533	0.95	1.638
SGD	0.9522	0.94	0.96	0.95	1.621
Adadelta	0.8303	0.7733	0.7867	0.7767	2.432
Adamax	0.9638	0.96	0.9433	0.9567	1.3818
Ftrl	0.6722	0.5767	0.8067	0.7033	1.8559

Significant values are in bold.

**Figure 9:**
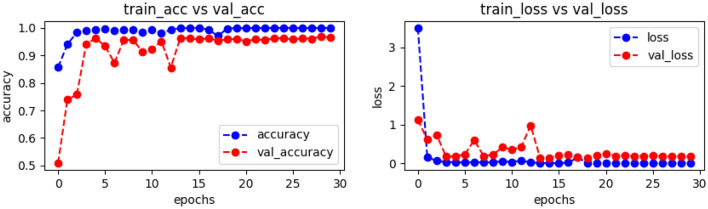
Outcomes of training accuracy and validation accuracy (left), as well as training loss and validation loss (right) on dataset_1.

Also, the proposed model was evaluated using binary classification of COVID-19 X-ray images and three classes consisting of coronavirus, normal, and viral pneumonia patients. The objective of this study was to assess the effectiveness of the Custom-CNN model in examining various relationships, including coronavirus and viral pneumonia, normal and viral pneumonia, and coronavirus and normal, as well as the associations among the three classes of coronavirus, normal, and viral pneumonia. In the experimental setup, a total of 1,345 chest X-ray images of pneumonia patients, 10,192 normal cases, and 3616 coronavirus-infected chest X-ray images were utilized. The outcomes were evaluated using various performance metrics such as accuracy, precision, recall/sensitivity, F1-score, and test loss, as shown in Table 8. The experimental results demonstrated that the proposed method achieved optimal classification results for the three classes, with accuracy, precision, recall/sensitivity, F1-score, and test loss values of 98.19, 97.67, 0.9833, 97.33, and 0.073, respectively. These results indicate that the Custom-CNN effectively handled the datasets, even in the case of imbalanced data, and achieved optimal outcomes for multi-class problems. Specifically, the proposed method exhibited superior classification results for COVID and normal images, with accuracy, precision, recall/sensitivity, F1-score, and test loss values of 98.55, 98.5, 0.98, 0.98, and 0.0441, respectively. Conversely, for COVID and viral pneumonia images, the suggested method yielded accuracy, precision, recall/sensitivity, F1-score, and test loss values of 99.50, 99, 99.5, 0.99.5, and 0.0306, respectively. Similarly, the proposed method achieved higher classification results for normal and viral pneumonia images, with accuracy, precision, recall/sensitivity, F1-score, and test loss values of 99.35, 99.5, 0.97, 98.5, and 0.0562, respectively. In conclusion, the findings of this study demonstrate that the Custom-CNN model accurately and rapidly identifies COVID-19 from chest X-ray images. To mitigate the risk of bias, a large dataset of COVID-19 cases was employed, and extensive preprocessing techniques were applied to ensure appropriate inputs to the CNN architecture. Figure 9 illustrates the training, validation accuracy, and validation loss for the different classes. In this figure, one may observe certain sudden small value changes (peaks) in the validation accuracy and validation loss. Such occurrences are common when there is a mismatch between the distribution or characteristics of the training and validation images. This mismatch is a result of the random selection process used for training and validation.

**Table 8:** Results of Custom-CNN model with different classes.

Cases	Classes (No.)	# of Images	Accuracy	precision	Recall/ sensitivity	f1-score	Test loss
1	COVID, Normal, Viral Pneumonia (3)	15,153	98.19	97.67	0.9833	97.33	0.073
2	COVID, Normal (2)	13,808	98.55	98.5	98	98	0.0441
3	COVID, Viral Pneumonia (2)	4961	99.50	99	99.5	99.5	0.0306
4	Normal, Viral Pneumonia (2)	11,537	99.35	99.5	97	98.5	0.0562

**Figure 10:**
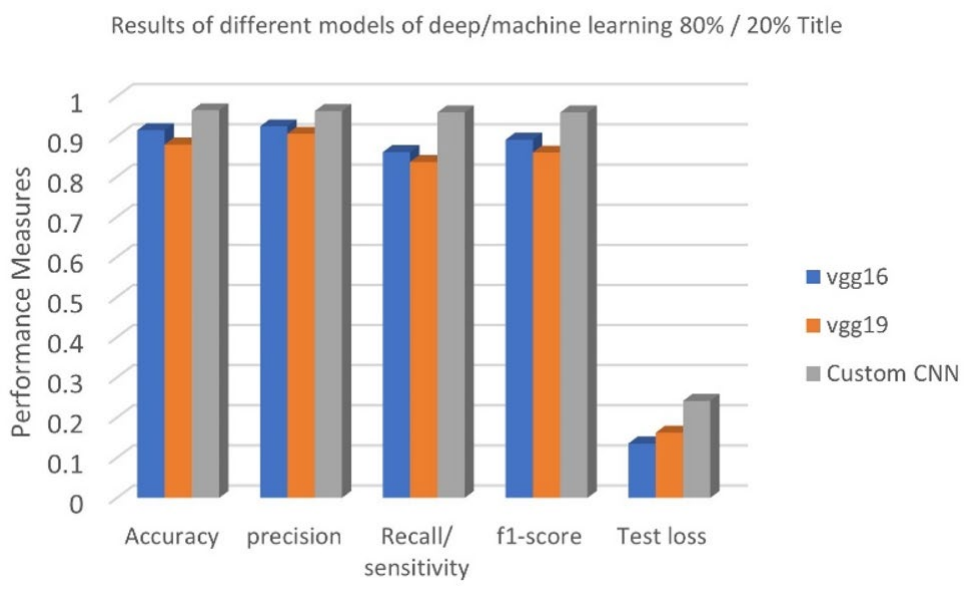
Measures for the different deep learning methods on dataset_1.

In this part, the effectiveness of the proposed Custom-CNN model in detecting COVID-19 images was evaluated using two deep learning techniques, namely vgg16 and vgg19, after determining the optimal parameter values for the Custom-CNN. The results demonstrated that the suggested model outperformed the other two approaches. Table 9 presents the outcomes for dataset_1 using various deep learning algorithms. Both Table 9 and Fig. 10 illustrate that the Custom-CNN achieved the highest classification accuracy of 0.9819, while vgg16 and vgg19 achieved accuracies of only 0.9159 and 0.88, respectively. Accuracy measures the percentage of positive samples that a model correctly identifies as positive samples. Additionally, Table 9 reveals that the Custom-CNN achieved the highest precision value of 0.9767, surpassing the precision values of vgg16 (0.9253) and vgg19 (0.9067). The Custom-CNN also attained the highest Recall/Sensitivity value of 0.9833, while vgg16 and vgg19 achieved Recall/Sensitivity values of 0.8612 and 0.8367, respectively. The F1-score is a suitable metric when seeking a technique that strikes a balance between precision and recall and provides a better measure of misclassified instances than accuracy. According to Table 9, the Custom-CNN obtained the highest F1-score of 0.9733, while vgg16 and vgg19 achieved scores of 0.8929 and 0.86, respectively. This F1-score result indicates that the Custom-CNN model performed better even when dealing with imbalanced class distributions, which is a common characteristic of real-world medical datasets.

**Table 9:** Results of applying the different models of deep learning on dataset_1.

Measure	Accuracy	Precision	Recall/ sensitivity	F1-score	Test loss
vgg16	0.9159	0.9253	0.8612	0.8921	0.1355
vgg19	0.88	0.9067	0.8367	0.86	0.1624
Custom-CNN	**0.9819**	**0.9767**	**0.9833**	**0.9733**	**0.073**

Significant values are in bold.

Furthermore, we conducted a comparison between our proposed model and vgg16 and vgg19 in terms of the required time. As shown in Table 10, our model demonstrated a significantly shorter time of 440s, compared to 3715s for vgg16 and 2899s for vgg19. It is worth noting that our proposed model has a smaller number of variables, with 9,701,571, in contrast to 14,714,688 for vgg16 and 20,024,384 for vgg19. However, when considering the ratio and proportion, our proposed model exhibits higher efficiency, as it requires less time.

**Table 10:** Differences in deep learning model training times using dataset_1.

	Training time	Average time/per step	Total parameters	Trainable parameters	Non-trainable parameters
vgg16	3715 s	325.76 ms	14,714,688	14,714,688	0
vgg19	2899 s	254.13 ms	20,024,384	20,024,384	0
Custom-CNN	440 s	38.03 ms	9,701,571	9,700,227	1344

To determine whether the suggested model is superior to the others, we compared our findings with those of other studies in the literature in this section’s final paragraph. Table 11 compares the metrics of the suggested approach to specifics from the literature, and displays that our results are better than those of others. These are the ones we compare ourselves to, some of whom used the same dataset as ours in our study, while others did not. Of course, we cannot achieve a fair comparison with those who used dataset different from ours, but it is a good indicator that can enlighten us about the performance level of our proposed algorithm in this research. As observed from the results shown in Table 11, none of them outperformed our proposed algorithm’s results, whether the dataset used was the same as our algorithm as in^29,51–56^ (highlighted in bold font in terms of the number of images) or different, as in the referenced studies^33,38–48,62^.

**Table 11:** The proposed Custom-CNN model is compared to many state-of-the-art deep learning models constructed using X-ray images to identify COVID-19 using three classes.

Study [reference #]	Year	Method proposed	Number of images	Performance (%)
Ozturk^33^	2020	DarkCOVIDNet	1125	Accuracy: 87.02 Precision: 89.96 Recall/sensitivity: 85.35 F1-score: 87.37 Test loss: 0.342
Apostolopoulos^38^	2020	deep CNNs	1442	Accuracy: 94.72 Precision: – Recall/sensitivity: 98.66 F1-score: – Test loss: –
Nishio^39^	2020	CADx-VGG16	1248	Accuracy: 83.68 Precision: 0.8842 Recall/sensitivity: 0.8936 F1-score: − 0.8889 Test loss: 0.4682
Wang^36^	2020	Tailored CNN	13,975	Accuracy: 93.3 Precision: 94.97 Recall/sensitivity: 96.92 F1-score: 95.93 Test loss: –
Li and Zhu^15^	2020	DenseNet	555	Accuracy: 88.9 Precision: 89.01 Recall/sensitivity: 97.59 F1-score: 93.1 Test loss: –
Oh et al.^41^	2020	Patch-based CNN	15,043	Accuracy: 88.9 Precision: 84.4 Recall/sensitivity: 85.9 F1-score: 84.4 Test loss: –
Nigam^42^	2021	EfficientNet	16,634	Accuracy: 93.48 Precision: 96.46 Recall/sensitivity: 96.46 F1-score: 96.46 Test loss: –
Zargari Khuzani^43^	2021	ML	420	Accuracy: 94.05 Precision: 96.24 Recall/sensitivity: 1 F1-score: 97.15 Test loss: 0.22
Gour^44^	2022	Stacked CNN: VGG19, Xception, Softmax classifier	3040	Accuracy: 97.27 Precision: 97.36 Recall/sensitivity: 97.62 F1-score: 97.5 Test loss: –
Karacı^45^	2022	Cascade VGGCOV19-NET	1125	Accuracy: 97.16 Precision: 98.53 Recall/sensitivity: 98.3 F1-score: 99.01 Test loss: –
Gour^46^	2022	UA-ConvNet: EfficientNet-B3, MC-dropout	3040	Accuracy: 97.67 Precision: 97.87 Recall/sensitivity: 98.15 F1-score: 97.99 Test loss: –
Kanwal^47^	2022	2dCNN-BiCuDNNLSTM	6863	Accuracy: 88.06 Precision: 92.33 Recall/sensitivity: 91.66 F1-score: 91.33 Test loss: 0.0999
Chong^51^	2021	ML	**15,153**	Accuracy: 90.87 Precision: – Recall/sensitivity: – F1-score: – Test loss: –
Sharma^52^	2023	Conv-CapsNet	**15,153**	Accuracy: 96.47 Precision: – Recall/sensitivity: – F1-score: – Test loss: –
Nikolaou^53^	2021	EfficientNetB0	**15,153**	Accuracy: 95 Precision: – Recall/sensitivity: 90 F1-score: – Test loss: –
Aslan^29^	2022	ML	**15,153**	Accuracy: 98.05 Precision: – Recall/sensitivity: – F1-score: – Test loss: –
Verma^54^	2022	CNN	**15,153**	Accuracy: 98 Precision: 98 Recall/sensitivity: 96 F1-score: 97 Test loss: –
Akinyelu^55^	2023	CapsNetCovid	**15,153**	Accuracy: 94.72 Precision: 93.86 Recall/sensitivity: 92.95 F1-score: 93.39 Test loss: –
Kavya^56^	2022	CNN(VGG16)	**15,153**	Accuracy: 89.34 Precision: 89 Recall/sensitivity: 89 F1-score: 89 Test loss: –
Kavya^56^	2022	CNN(ResNet50)	**15,153**	Accuracy: 91.39 Precision: 91.3 Recall/sensitivity: 90 F1-score: 91 Test loss: –
Proposed model		Custom-CNN	**15,153**^55^	Accuracy: 98.19 Precision: 97.67 Recall/sensitivity: 0.9833 F1-score: 97.33 Test loss: 0.073

The bold font highlights the number of images, indicating the usage of the same dataset.

### Evaluation of the Custom-CNN using dataset_2

**Figure 11:**
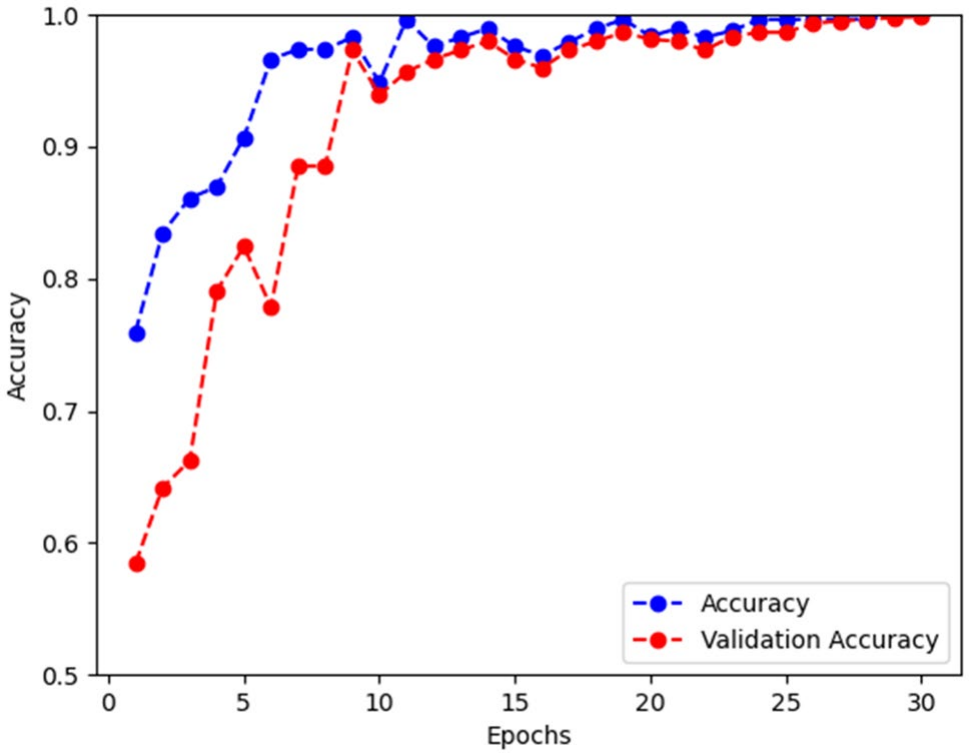
Outcomes of training accuracy and validation accuracy on dataset_2.

In this section, after determining the optimal parameters for the proposed Custom-CNN model in the previous section, our objective was to further validate the effectiveness of the model by applying it to analyze dataset_2, a new set of images. Figure 11 depicts the progression of the proposed model during the training phase of Dataset_2. The outcomes of the suggested model, compared to the latest findings, are presented in Table 12, where the data is divided into training and testing sets with proportions of 80% and 20% respectively. It should be noted that some of the compared results utilized the same dataset as ours, as mentioned in^59^, while others employed different datasets, as referenced in ^28,29,50–56,62,63^. Although a fair comparison cannot be made with those who used different datasets, this comparison serves as a valuable indicator to evaluate the performance of our proposed algorithm in this research. Upon examining the results displayed in Table 12, our proposed model achieved outstanding results with a classification accuracy of 99.8%, precision of 99.9%, recall/sensitivity of 99.7%, F1-score of 99.8%, and a test loss of 0.0710, surpassing other state-of-the-art competitors.

**Table 12:** Results of employing the Custom-CNN model on dataset_2 are compared to state-of-the-art research for binary classes (COVID-19/Normal).

Author name [reference #]	Year	Method proposed	Number of images	Performance (%)
Ozturk^33^	2020	DarkCOVIDNet	625	Accuracy: 98.08 Precision: 98.03 Recall/ sensitivity: 95.13 F1-score: 96.51 Test loss: 0.0992
Apostolopoulos^38^	2020	deep CNNs	1442	Accuracy: 96.78 Precision: - Recall/ sensitivity: 98.66 F1-score: – Test loss: –
Rahman^28^	2022	QCovSML	2656	Accuracy: 93.34 Precision: 92.02 Recall/ sensitivity: 95.59 F1-score: 93.73 Test loss: –
Cabitza^63^	2021	meta-validation ML method	2656	Accuracy: 77 Precision: 76 Recall/ sensitivity: 60 F1-score: 63 Test loss: 2.327
Narin^62^	2021	ResNet50	3141	Accuracy: 96.1 Precision: 84.2 Recall/ sensitivity: 91.8 F1-score: 83.5 Test loss: 0.1832
Medhi^46^	2020	Deep CNN	357	Accuracy: 93 Precision: 98.79 Recall/sensitivity: 93.98 F1-score: 96.32 Test loss:
Abbas^47^	2021	DeTraC deep CNN	1763	Accuracy: 93.1 Precision: – Recall/ sensitivity: 100 F1-score: – Test loss: –
Bargshady^48^	2022	CycleGAN-Inception	9544	Accuracy: 94.2 Precision: 95.48 Recall/ sensitivity: 96.2 F1-score: 95.84 Test loss: –
Sahin^50^	2022	CNN_Model	13,824	Accuracy: 96.71 Precision: 94.25 Recall/ sensitivity: 92.78 F1-score: 93.51 Test loss: –
Khan^59^	2021	SVM	**340**	Accuracy: 94.12 Precision: Recall/ sensitivity: F1-score: Test loss:
Khan^59^	2021	CNN	**340**	Accuracy: 78.43 Precision: Recall/ sensitivity: F1-score: Test loss:
Proposed model		Custom-CNN	**340**	Accuracy: 99.8 Precision: 99.9 Recall/ sensitivity: 99.7 F1-score: 99.8 Test loss: 0.0710

The bold font highlights the number of images, indicating the usage of the same dataset.

## Conclusion

Chest X-rays were utilized in this study to diagnose COVID-19 and detect the presence of the coronavirus, aiming to address the issues related to the accuracy and time requirements of RT-PCR. Due to their lower cost compared to CT scans, chest X-rays were given more consideration in this study. Additionally, CT scans involve a higher level of ionizing radiation compared to X-rays. The proposed Custom-CNN model, which features an end-to-end structure and full automation, eliminates the need for human feature extraction. This approach can be particularly beneficial for countries heavily affected by COVID-19, as it addresses the shortage of radiologists. The comprehensive assessment conducted revealed that the analyzed chest X-ray images exhibited distinct patterns and bilateral alterations. However, the manual approach to COVID-19 detection using X-rays is challenging. Therefore, this study employed a deep learning-based methodology to automatically analyze chest X-rays. The performance of the process was evaluated through a thorough comparative analysis, with accuracy as the performance criterion. The results of the analysis demonstrated that the recommended model outperforms the other models. The only limitation of this study was the limited number of chest X-ray images that were reviewed.

## Data Availability

We used free available datasets, *dataset_1* from “https://www.kaggle.com/datasets/tawsifurrahman/COVID19-radiography-database” and *dataset_2* from “https://github.com/ieee8023/COVID-chestxray-dataset”.
